# Structural Characteristics and Properties of Cocoon and Regenerated Silk Fibroin from Different Silkworm Strains

**DOI:** 10.3390/ijms24054965

**Published:** 2023-03-04

**Authors:** Yeon Jin Kim, Seong Wan Kim, Kee Young Kim, Chang Seok Ki, In Chul Um

**Affiliations:** 1 Department of Biofibers and Biomaterials Science, Kyungpook National University, Daegu 41566, Republic of Korea; 2 National Institute of Agricultural Sciences, RDA, Wanju 55365, Republic of Korea; 3 Department of Agriculture, Forestry and Bioresources, Seoul National University, Seoul 08826, Republic of Korea; 4 Research Institute of Agriculture and Life Science, Seoul National University, Seoul 08826, Republic of Korea

**Keywords:** silkworm strain, cocoon, silk fibroin, degumming, structural characteristics, mechanical properties

## Abstract

Silk has attracted the attention of researchers as a biomedical and cosmetic material because of its good biocompatibility and cytocompatibility. Silk is produced from the cocoons of silkworms, which have various strains. In this study, silkworm cocoons and silk fibroins (SFs) were obtained from ten silkworm strains, and their structural characteristics and properties were examined. The morphological structure of the cocoons depended on the silkworm strains. The degumming ratio of silk ranged from 22.8% to 28% depending on the silkworm strains. The highest and lowest solution viscosities of SF were shown by 9671 and 9153, respectively, showing a 12-fold difference. The silkworm strains of 9671, KJ5, and I-NOVI showed a two-fold higher work of ruptures for the regenerated SF film than 181 and 2203, indicating that the silkworm strains considerably influence the mechanical properties of the regenerated SF film. Regardless of the silkworm strain, all silkworm cocoons showed good cell viability, making them suitable candidates for advanced functional biomaterials.

## 1. Introduction

Silk is a protein-based fiber with a crystalline structure and is composed of two biopolymers: fibroin and sericin. It has been used as the best textile material for a long time. Recently, silk has been studied and used as a promising biomedical as well as cosmetic material because of its good biocompatibility [1,2], excellent cytocompatibility [3,4], and good biodegradability [5,6]. The biomedical and cosmetic applications of silk include wound dressing [7], drug delivery [8], artificial heart valves [9], nerve conduits [10], tympanic membranes [11,12], membranes for guided bone regeneration [13,14], and cosmetic mask packs [15].

For these applications, silk needs to be fabricated into various forms, including fiber [16,17], film [18,19], nanoweb [20,21], nonwoven fabric [22,23], sponge [24,25], bead [26], and gel [27,28,29]. Furthermore, these applications require various performances of the silk material. To achieve the performance of silk, researchers add foreign materials into the silk using facile methods [30,31,32,33]. However, in some cases, the addition of foreign material may deteriorate the unique properties of silk. To prevent this deterioration, a regenerated silk fibroin (SF) can be prepared under different conditions rather than introducing foreign materials. Moreover, their effect on the structure and properties of SF has been examined to know the best performance of SF only. The preparation condition includes the selection of solvents [18,34], degumming conditions [35], sericin content [19,36,37], control of molecular weight(s) (MW) [25,28,38,39], post drawing [39,40], and the use of silk nanofibril [41].

Recently, extensive studies have been conducted on the effect of silkworm variety on the structure and properties of silk which showed that the structures and properties of SF and sericin differed depending on the silkworm variety [42,43]. Furthermore, the electrospinning performance of SF [44], the character of raw sericin in silk [45], and the fabrication of the nonwoven fabric of natural silk [46] depended on the silkworm variety.

Breeding can be used to produce unlimited silkworm strains. Among the silkworm strains, a few have been selected to use commercially. This is known as “silkworm variety”. In recent years, several silkworm varieties and strains have been used in related studies. However, there are still many silkworm strains that should be explored further. Furthermore, various properties of silk from the different silkworm strains have not yet been studied in detail.

In the present study, we choose ten silkworm strains showing good growing performance, good hatchability, high pupation rates (93–99%), and high cocoon shell ratios (21–26%) among thousands of silkworm strains in Korea. We examined the structural characteristics and properties of the silkworm cocoons, degummed silk (SF), and regenerated SF film obtained from the different strains.

## 2. Results and Discussion

### 2.1. Structural and Morphological Characteristics of Cocoons and Degummed Silk

Table 1 shows the external features and morphological structures of the cocoons from the different silkworm strains. Depending on the silkworm strain, the cocoons exhibited different oval shapes. The outer diameter of the cocoon fiber ranged from 26.5 to 29.4 µm, and, in the case of the degummed silk, ranged from 12.4 to 15.3 µm depending on the silkworm strain. This indicates that the diameters of the fibroin strand (degummed silk) and silk filament (in the raw cocoon) vary depending on the silkworm strain. Moreover, the diameter of silk in the cocoon becomes almost half that of the degummed silk, considering the two fibroin strands come out of a silk filament via degumming.

The thickness of the silkworm cocoons ranged from 0.52 to 1.21 mm depending on the silkworm strain. The different morphologies of silkworm cocoons of the different strains are consistent with the results reported in previous studies [42,47,48].

Notably, the degummed silk showed different morphologies depending on the silkworm strains. When the silk is over-degummed, microfibrils are generated on the fibroin surface [37,41]. Interestingly, the degree of microfibril generation in the degummed silk filament depended on the silkworm strains. Silkworm strains 2607, 9153, and KJ5 showed more microfibrils than the others on the degummed silk filaments. This implies that the silk filament is damaged differently by degumming depending on the silkworm strain. Considering that the microfibril generation of silk is caused by the physical damage to silk fibroin filaments by the degumming agent and the physical force of boiling water, it is assumed that the dissolution of covering material (i.e., sericin), and the resistance of fibroin against those two factors, are different depending on the silkworm strain.

### 2.2. Degumming and Molecular Conformation of Silks

**Figure 1:**
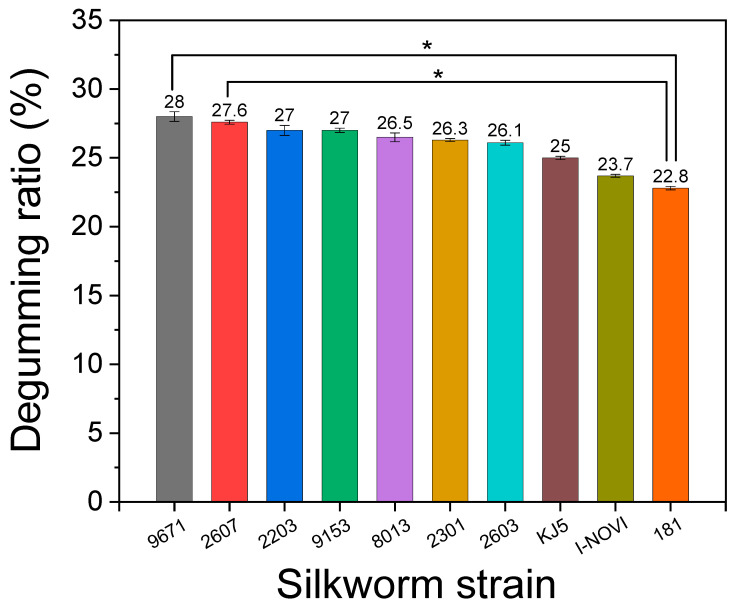
The degumming ratio of the silkworm cocoons produced from different silkworm strains (* *p* < 0.05, *n* = 3).

Figure 1 shows the degumming ratio (the ratio of the amount of sericin and nonprotein materials removed from silk during degumming) of cocoons from different silkworm strains. The degumming ratio of silk varies from 22.8% to 28% depending on the silkworm strain, indicating that the degumming of silk is dependent on the silkworm strain. In particular, strains 181 and I-NOVI showed significant differences compared with strains 9671 and 2607, respectively. Kim and Um also reported that the degumming ratio of silk ranged from 24.1% to 30.1% depending on the silkworm variety [45]. Therefore, the amount of sericin varies depending on the silkworm strain and variety, and, depending on the application, we can alter the amount of sericin and fibroin in silk by choosing the appropriate silkworm strain and variety. This means that when we need more sericin, we can use the 9671 or 2607 silkworm strain, and when we need fibroin only, we can use the 181 silkworm strain.

**Figure 2:**
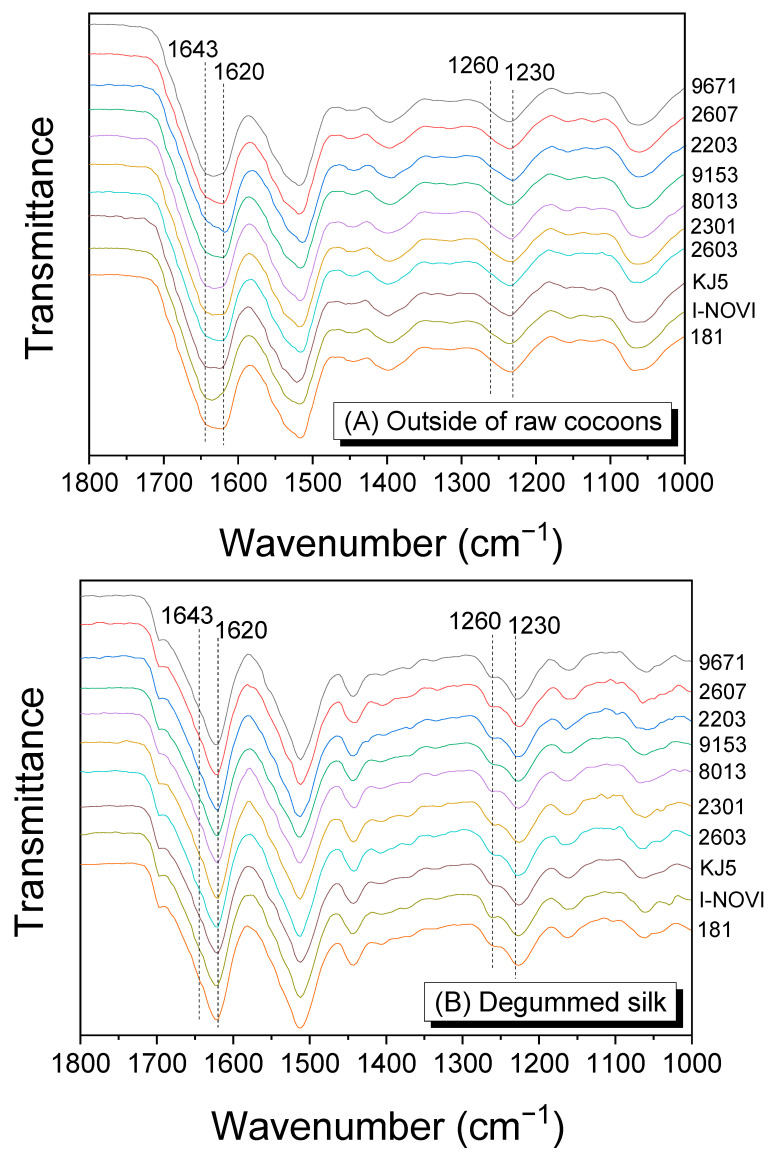

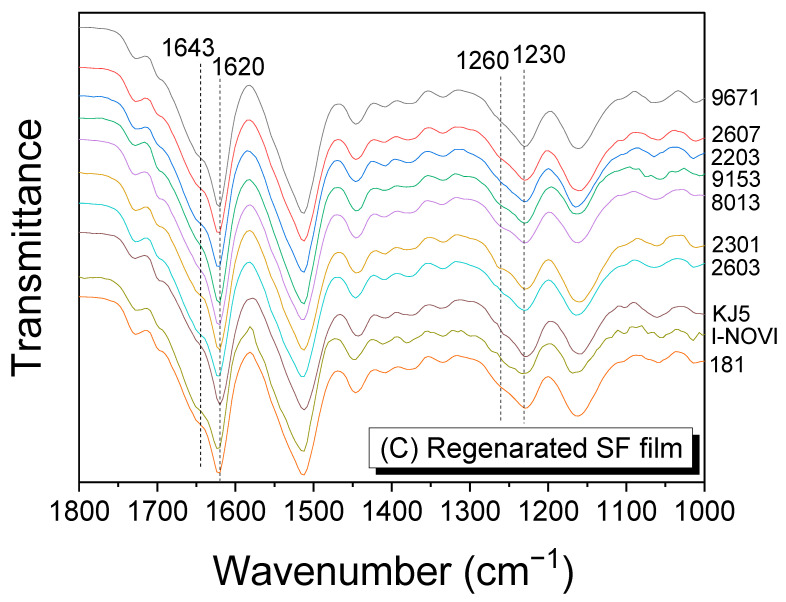
The ATR–FTIR spectra of (**A**) silk outside of silkworm cocoons, (**B**) degummed silk (SF), and (**C**) regenerated SF film from different silkworm strains.

FTIR spectroscopy was used to examine the molecular conformation and crystallinity of the silk materials because they determine the properties of silk, including mechanical properties [19,36], post-drawing character [39,40], and moisture regain [19]. Figure 2 shows the FTIR spectra of raw silk (outside of the cocoon), degummed silk, and the regenerated SF. The cocoons showed IR absorptions at 1643 and 1620 cm^−1^ in the amide I band, attributed to the random coil and β-sheet conformation, respectively [18,41]. The intensity of IR absorptions at 1643 and 1620 cm^−1^ differed depending on the silkworm strain, indicating that the crystallinity of silk differed depending on the silkworm strain. In the amide III band, IR absorption peaked at 1230 cm^−1^, corresponding to the random coil conformation.

Degummed silks (Figure 2B) showed a strong IR peak at 1620 cm^−1^ and a shoulder at 1260 cm^−1^ attributed to β-sheet conformation, indicating that the crystallinity of silk is improved by degumming. This is because less crystalline sericin (than fibroin) is removed from the silk by degumming [35]. Regenerated SF films (Figure 2C) had a strong IR peak at 1620 cm^−1^ and a weak shoulder at 1643 cm^−1^, indicating that they crystallized more than raw silks during formic acid casting [18,49].

**Figure 3:**
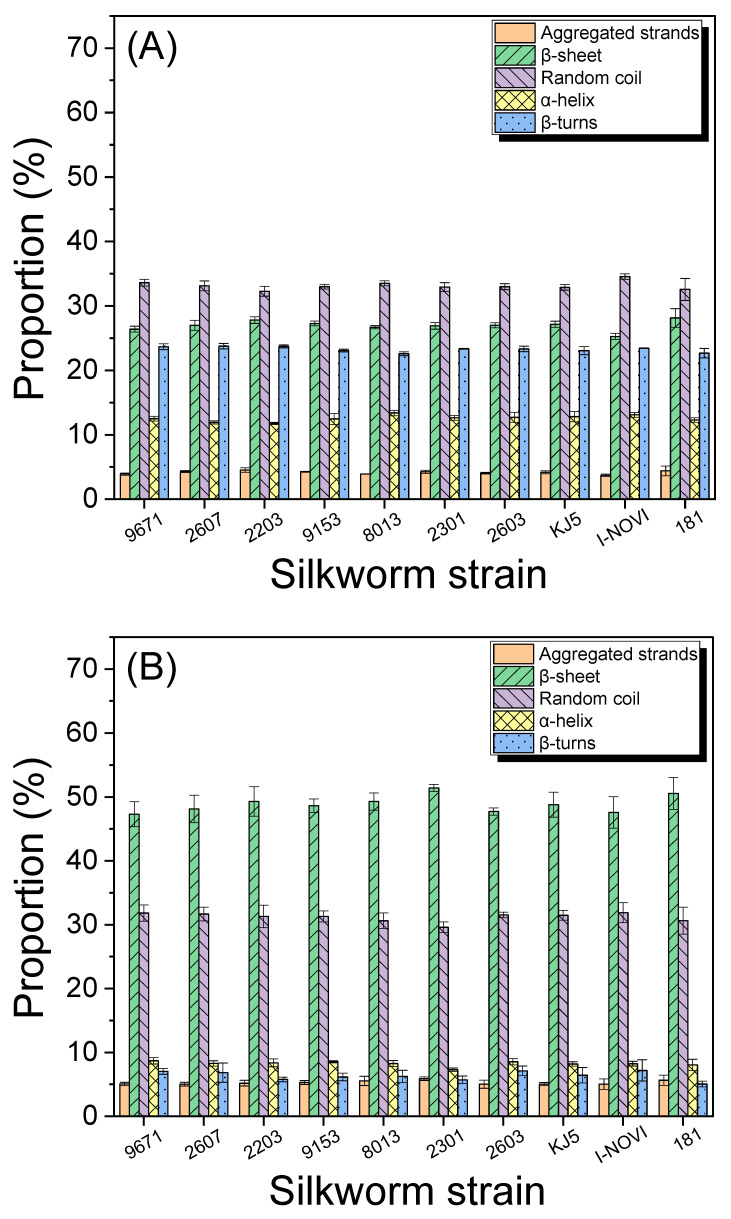

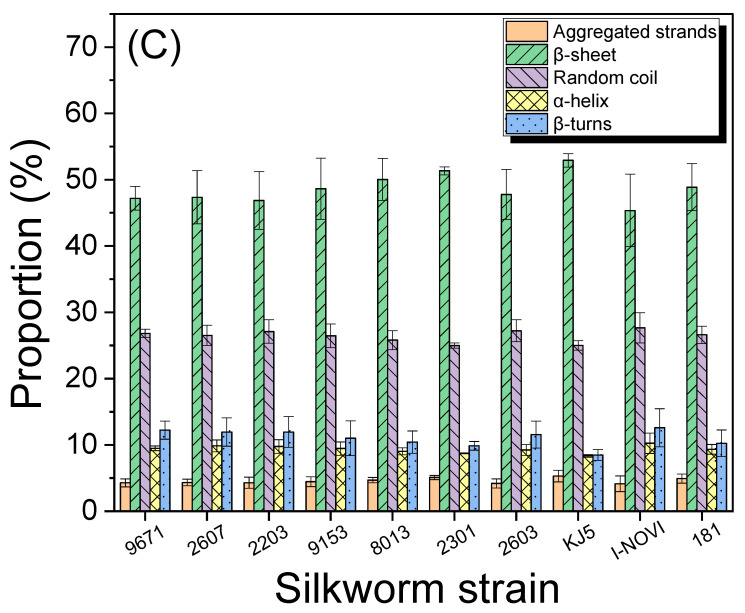
The proportion of the conformations of (**A**) silk outside of silkworm cocoons, (**B**) degummed silk (SF) and (**C**) regenerated SF film from various silkworm strains. Proportions of the molecular conformations were estimated using the deconvolved amide Ⅰ band. The error bars represent the standard deviation. (*n* = 3).

To quantitatively examine the molecular conformation of silks from different silkworm strains, the amide I band (1600–1700 cm^−1^) of silks was deconvolved, and the results are shown in Figure 3. The silk cocoons (raw silk) (Figure 3A) showed 25–30% of β-sheet and 30–35% of random coil conformations. In contrast, degummed silks (Figure 3B) and regenerated SF films (Figure 3C) showed 45–55% of β-sheet and 25–35% of random coil conformations. This confirms that the degummed silks and regenerated SF films are more crystalline than the cocoons, as shown in Figure 2. However, there were no statistical differences in the composition of the secondary structure among different silkworm strains. This indicates that the silkworm strains have only a minimal effect on the secondary structure of silk proteins.

### 2.3. Solution Viscosity of Regenerated SF

**Figure 4:**
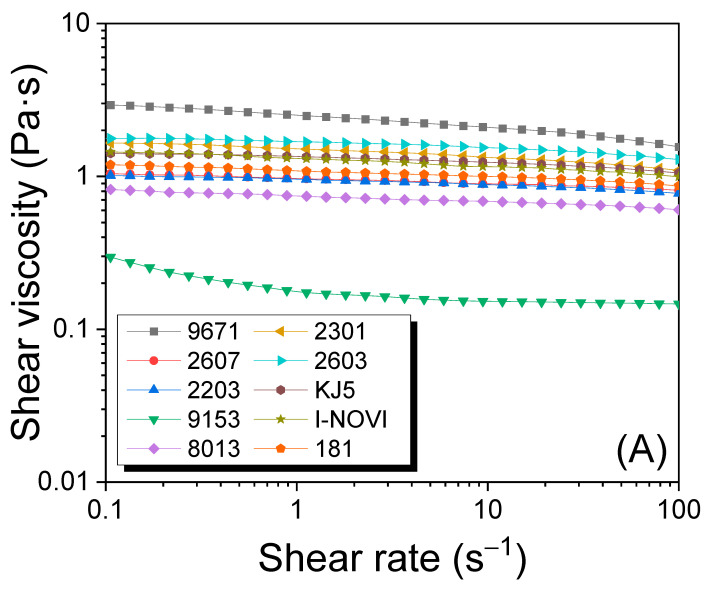

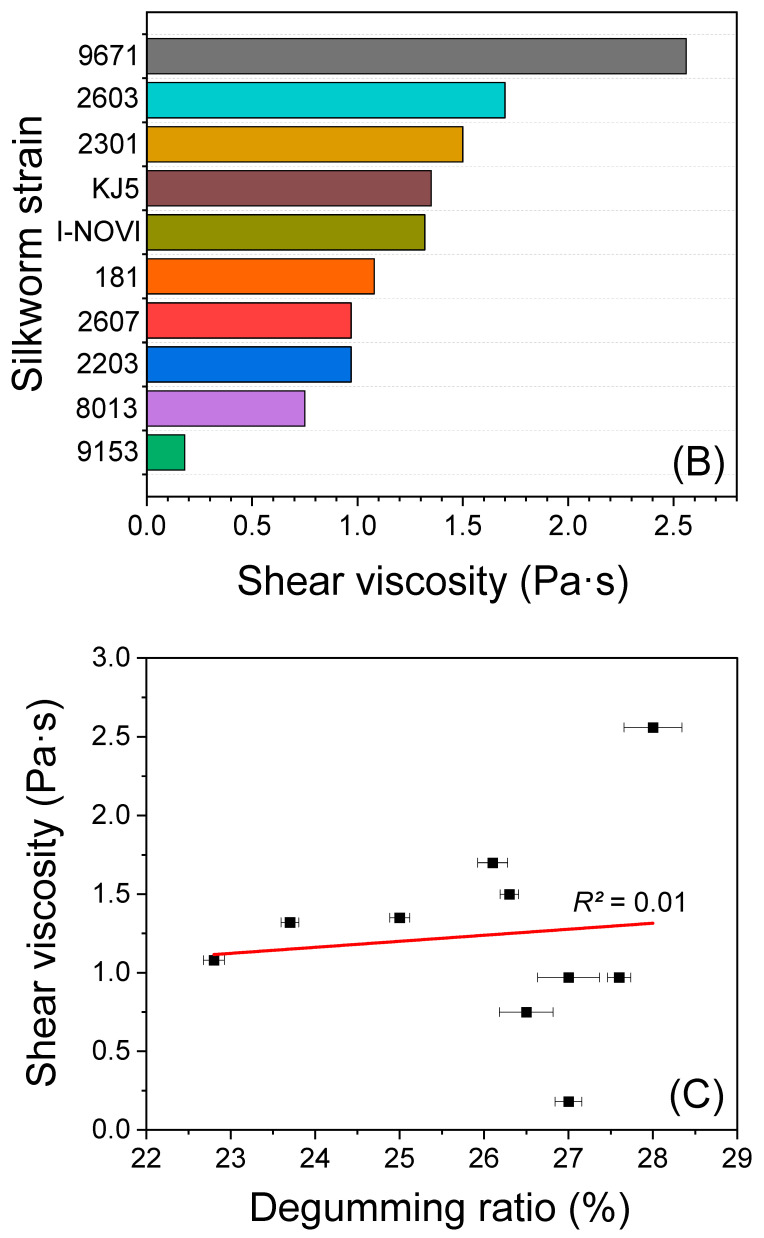
(**A**) The steady-state flow, (**B**) shear viscosity at 1 s^−1^ of 10% (*w*/*w*) regenerated SF formic acid solutions from different silkworm strains, and (**C**) correlation of degumming ratio of degummed silk and shear viscosity at 1 s⁻^1^ of 10% (*w*/*w*) regenerated SF formic acid solutions from different silkworm strains.

The molecular weight (MW) of silk strongly affects the properties of the silk materials [25,38,50,51]. Therefore, the measurement of MW is an important parameter in the study of silk materials. The MW of silk has been mainly evaluated using sodium dodecyl sulfate–polyacrylamide gel electrophoresis (SDS–PAGE) [49] and liquid chromatography [25,42,50,52]. However, evaluating the MW of silk precisely using SDS–PAGE is challenging owing to the broad bands that appear during the measurement. Moreover, obtaining data using liquid chromatography is highly complex and challenging, although it provides extremely precise results. However, the viscosity measurement of the silk formic acid solution using a rheometer is straightforward and shows a good correlation with the MW results from liquid chromatography [25,42,50]. Therefore, the viscosity of the regenerated SF solution from different silkworm strains was measured, and the results are shown in Figure 4. Most silk formic acid solutions showed a Newtonian fluid behavior, whereas silkworm strain 9153 exhibited a slight shear thinning in the low shear rate region (0.1–1 s^−1^). Therefore, the viscosity of SF solutions depends on the silkworm strain. The silkworm strain 9671 showed the highest viscosity at 1 s^−1^ (2.5 Pa∙s), and 9153 displayed the lowest viscosity (0.2 Pa∙s). This implies that the silkworm strain strongly affects the MW of SF. Chung et al. also reported that the MW and solution viscosity of SF differed depending on the silkworm variety [42].

However, the exact reason behind the different MWs of SF from different silkworm strains cannot be elucidated by the present study; it is assumed that the interactions of fibroin and sericin during degumming and dissolution are different depending on the silkworm strain. The molecular degradation of SF occurs during degumming and dissolution, decreasing the MW of SF [35,50,53,54]. A previous study [42] reported that SF from different silkworm varieties showed the main MW peak at the same MW position and different amounts of lower MW regions. This indicates that the molecular degradation of SF occurs differently depending on the silkworm variety. The sericin may prevent SF degradation during degumming, depending on the silkworm strain. It has been reported that the binding character of raw sericin [45] and the mechanical properties of sericin film [43] are different depending on the silkworm variety. Considering the variations in sericin characteristics from different silkworm varieties and strains, sericin dissolution may vary, affecting the molecular degradation of SF during the degumming process. Furthermore, the SF dissolution characteristics from different silkworm strains might be responsible for the differences in the molecular degradation of SF during dissolution.

For reference, there is no correlation (*R*^2^ = 0.01) between the amount of sericin in silk (i.e., degumming ratio) and the MW of fibroin, indicating that the amount of sericin in silk does not affect MW of SF (Figure 4C).

### 2.4. Mechanical Properties of Regenerated SF Film

**Figure 5:**
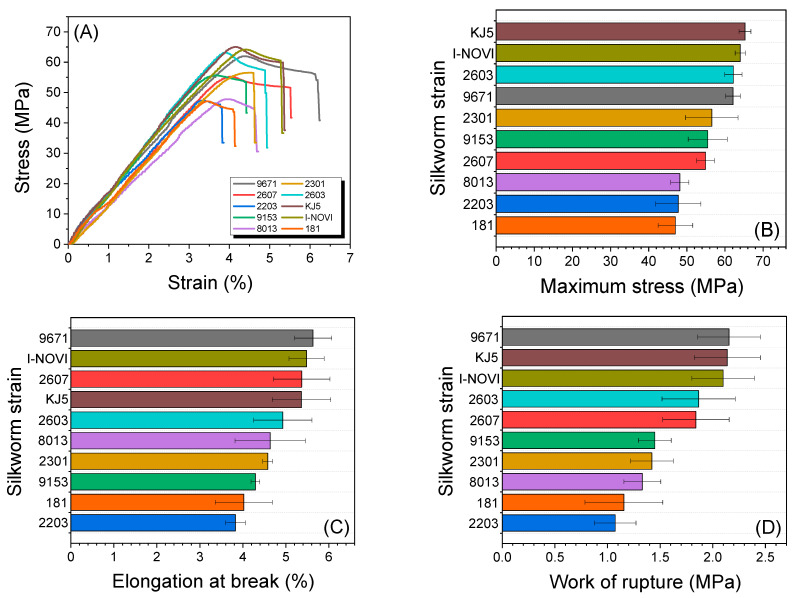
(**A**) The representative stress-strain curve, (**B**) maximum stress, (**C**) elongation at break, and (**D**) work of rupture of the regenerated SF film from different silkworm strains (*n* = 7).

Figure 5 shows the mechanical properties of the regenerated SF film from different silkworm strains. The stress in all the regenerated SF films increased linearly up to a certain point with increasing strain, then decreased marginally before the break (Figure 5A). As shown in Figure 5B, the maximum stress in the SF film varies from 47 MPa to 65 MPa, depending on the silkworm strain. The silkworm strain KJ5 showed the highest maximum stress, whereas the silkworm strain 181 displayed the lowest maximum stress. The elongation at break also differed depending on the silkworm strain, ranging from 3.8% to 5.6%. The silkworm strain 9671 showed the highest elongation at break, while the silkworm strain 2203 exhibited the lowest elongation. The work of rupture ranged from 1.1 MPa to 2.2 MPa. The silkworm strains 9671, KJ5, and I-NOVI showed the highest work of rupture, and the silkworm strains 181 and 2203 displayed the lowest work of rupture. Overall, the regenerated SF films of the silkworm strains 9671, KJ5, and I-NOVI showed better mechanical properties than those of the other strains. This result demonstrates that the mechanical properties of the regenerated SF film depend on the silkworm strain.

**Figure 6:**
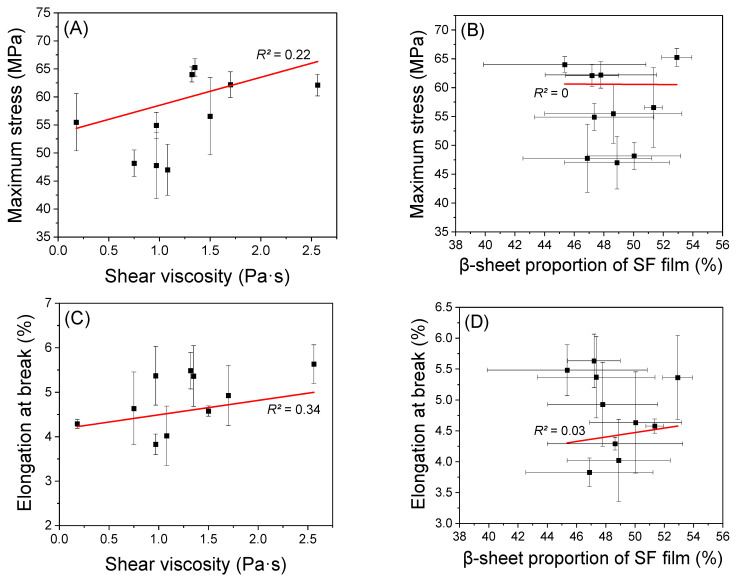

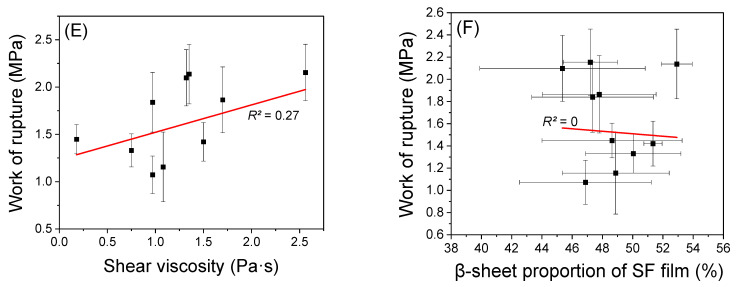
The relationship between the shear viscosity at 1 s^−1^ of the 10% (*w*/*w*) regenerated SF formic acid solutions/the β-sheet proportion (%) of the SF films and mechanical properties of the regenerated SF films produced from different silkworm strains; (**A**,**B**) maximum stress, (**C**,**D**) elongation at break, and (**E**,**F**) the work of rupture of the regenerated SF films.

Moreover, two factors may affect the mechanical properties of regenerated SF films: MW (solution viscosity) and crystallinity (the content of β-sheet conformation). Therefore, the β-sheet proportion and solution viscosity were plotted against the mechanical properties (the maximum stress, elongation at break, and work of rupture), and the results are shown in Figure 6. The β-sheet proportion showed an *R*^2^ of approximately zero, indicating that there was no effect on the mechanical properties of the SF. In contrast, the solution viscosity showed *R*^2^ of 0.22, 0.34, and 0.27 for the maximum stress, elongation at break, and work of rupture, respectively. Although these *R*^2^ values indicate a lower correlation between the solution viscosity and mechanical properties, it can be said that the solution viscosity affects the mechanical properties of SF very slightly. As the mechanical properties of SF films are determined by the combined effect of many factors, it is difficult to precisely examine the effects of all factors. However, it can be said that the solution viscosity (i.e., MW of SF) is a more dominant factor in influencing the mechanical properties of SF film than the β-sheet proportion in SF. Moreover, considering MW and crystallinity are the main factors influencing the mechanical properties of SF, as reported in the previous studies [19,36,38,51], it was surprising to find that the solution viscosity (*R*^2^ = 0.22–0.34) and β-sheet proportion (*R*^2^ = 0–0.03) showed little or no relationship with the mechanical properties of SF films. It is assumed that various unknown factors influence the mechanical properties of SF films, as well as MW and crystallinity. However, all the factors cannot be elucidated precisely in the present study.

### 2.5. Cytocompatibility of the Cocoon

**Figure 7:**
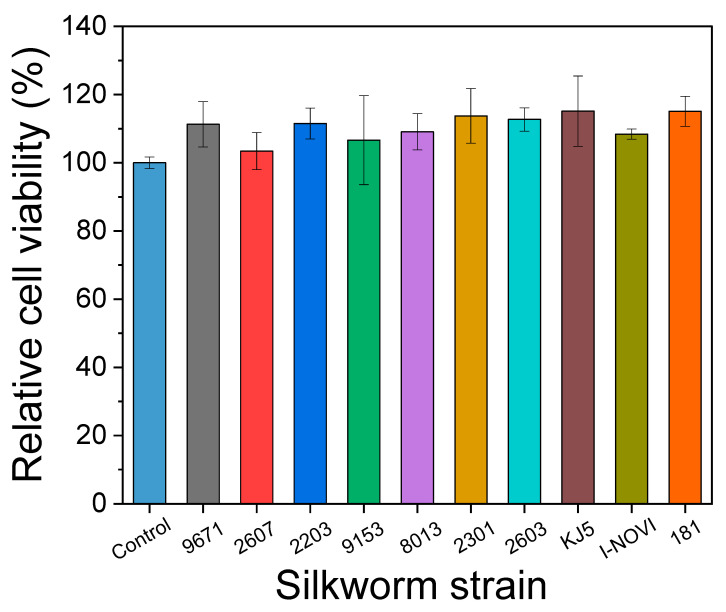
The relative cell viabilities of NIH3T3 cells treated with the extracts of cocoons produced from different silkworm strains (mean ± SD, *n* = 3). The one-way ANOVA test result indicated no significant differences among the compared groups.

**Figure 8:**
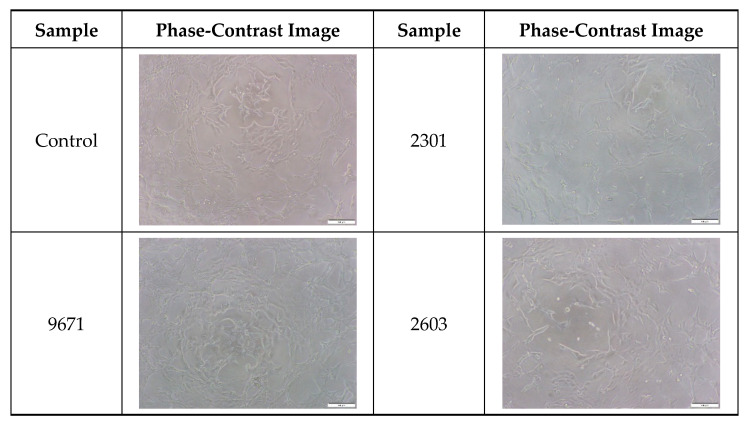

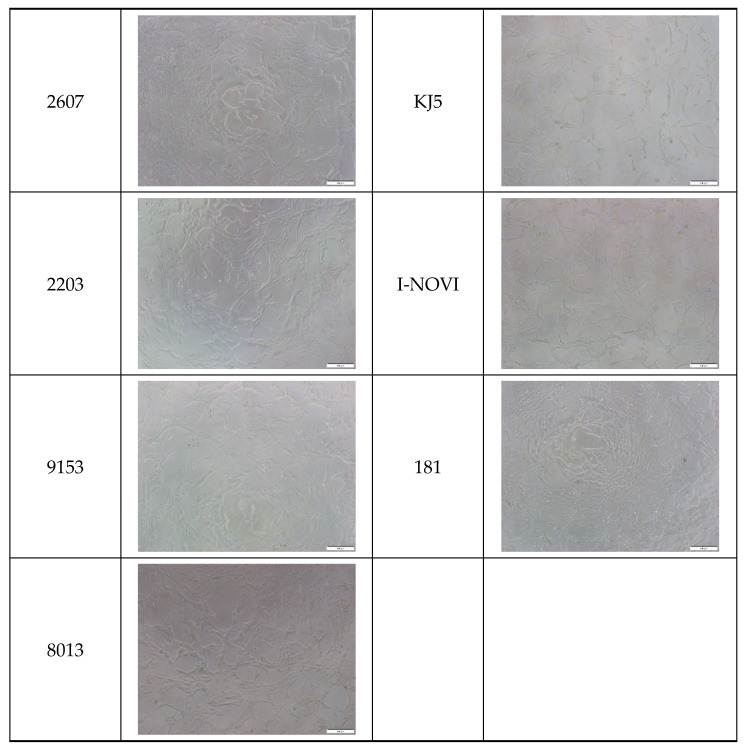
The phase-contrast images of NIH3T3 cells treated with the extracts of cocoons produced from different silkworm strains. The magnification bar in the images represents 100 μm.

As silk is used in biomedical and cosmetic applications, it is necessary to evaluate its cell viability. Therefore, cell viability tests of the cocoons from different silkworm strains were conducted, and the results are shown in Figure 7 and Figure 8. Figure 7 shows the relative cell viability of NIH3T3 cells treated with the extracts of silkworm cocoons. The relative cell viabilities were not statistically different for all groups, and no significant differences were shown compared to the control group (i.e., untreated cells). In addition, the cell morphology was not changed after the cocoon extract treatment (Figure 8). These observations indicated that the cocoons were not cytotoxic, regardless of the silkworm strains.

## 3. Materials and Methods

### 3.1. Materials

Ten different *Bombyx mori* silkworm strains (9671, 2607, 2203, 9153, 8013, 2301, 2603, KJ5, I-NOVI, and 181) were grown at the National Institute of Agricultural Sciences (Wanju, Republic of Korea). Silkworms were hatched under a temperature of 15–26 °C and a humidity of 75–80% with 16 h light and 8 h dark photoperiod conditions. They were reared at 25 °C and fed with mulberry leaves and an artificial diet. Silkworms were reared following Sericultural Experiment Guide as follows; 1st~3rd instar (25–26 °C temperature, 75–80% humidity covered with wax paper), 4th~5th instar (23–24 °C temperature, 65–75% humidity). Silkworms were fed three times a day with mulberry leaves [55]. Ten silk cocoon samples were produced from the ten silkworm strains.

### 3.2. Preparation of Regenerated SF Solution and Films

The preparation of regenerated SF solution and film has been reported in a previous study [42]. The cocoons were degummed in an aqueous solution containing 0.3% (*w*/*v*) sodium oleate and 0.2% (*w*/*v*) sodium carbonate at 100 °C for 1 h. The ratio of the cocoon and degumming solution was 1:25 (*w*/*v*). After degumming, the cocoons were rinsed thoroughly with water purified using a water purification system (RO50, Hana Science, Hanam, Republic of Korea) with a reverse osmosis membrane and then dried. The degummed silk was dissolved in a ternary solvent containing CaCl_2_/H_2_O/EtOH (1/8/2 molar ratio) at 85 °C for 30 min. The ratio of silk and ternary solvent was 1:20 (*w*/*v*). The regenerated aqueous SF solutions were obtained by dialyzing the dissolved SF solutions using a cellulose tube (MW cut-off = 12,000–14,000 Da) against circulating purified water for four days at room temperature. The regenerated aqueous SF solution was filtered and dried to obtain the regenerated SF powder. Then, the powder was dissolved in 98% formic acid to prepare the 10% (*w*/*w*) SF formic acid solution for rheological measurement, and 20 mL of a 2% (*w*/*w*) SF solution was poured into a 9 cm petri dish and dried under a hood at 25 °C to create the regenerated SF films.

### 3.3. Measurement and Characterization

The color and external features of the ten silkworm cocoon strains were photographed using a digital camera (iPhone 11, Apple Inc., Cupertino, CA, USA).

The morphologies of the silk cocoons and the degummed silk fibers were examined using a field emission-scanning electron microscope (FE-SEM, S-4800, Hitachi, Tokyo, Japan) [56,57]. The cocoons and degummed silk fibers were then coated with Pt–Pd before observation. The means and standard deviations of the silk filament diameters were obtained by measuring 30 silk filaments from the SEM images using an image analysis program (DIMIS-PRO 2.0, Siwon Optical Technology, Anyang, Republic of Korea).

The thicknesses of the cocoons were measured using a thickness gauge (MDC-25PXT, Mitutoyo, Kawasaki, Japan). Twenty different parts of cocoons for each silkworm strain were measured, and the average thicknesses were calculated based on these twenty measurements.

The following equation was used to calculate the degumming ratios: degumming ratio (%) = (1 − dry weight of degummed cocoons/dry weight of native cocoons) × 100 [58]. The dry weight of each cocoon was measured using a moisture analyzer (XM60, Precisa Gravimetric, Dietikon, Switzerland).

Fourier-transform infrared spectroscopy (FTIR, Nicolet 380, Thermo Fisher Scientific, Waltham, MA, USA) was employed in the attenuated total reflection mode to examine the molecular conformation of the outer part of raw cocoons, the degummed silk, and the regenerated SF film of different silkworm strains [59].

The proportions of the β–sheet and random coil conformation of the cocoons and SFs were determined by deconvoluting the amide I band (1600–1700 cm^−1^) of the cocoons and SFs using the Fourier self-deconvolution fitting method in the Origin 8.0 software to examine the effect of the silkworm strains on the conformational change in the cocoons and SFs [22,28,60].

The 10% (*w*/*w*) regenerated SF formic acid solution was used for the rheological measurements. The shear viscosity was measured using a Thermo-HAAKE rheometer (MARS III, Thermo Scientific, Karlsruhe, Germany) with a cone and plate geometry with a shear rate of 0.1–100 s^−1^ at 25 °C. The radius and angle of the cone were 60 mm and 1°, respectively [50].

To evaluate the mechanical properties of the regenerated SF film, the maximum stress, elongation at break, and work of rupture were obtained using a Universal Test Machine (OTT-03, Oriental TM, Ansan, South Korea) [23,42,61]. The tensile tests were performed using a 3 kgf load cell at an extension rate of 10 mm/min for the SF film. The length and width of the prepared film samples were 50 mm and 5 mm, respectively. The gauge length was 30 mm. All samples were preconditioned under the standard conditions (i.e., 20 °C and 65% relative humidity) for more than 24 h. Furthermore, the mechanical testing of the samples was carried out under standard conditions. Seven different SF film samples for each silkworm strain were used for the measurement. After the maximum and minimum values were obtained, the mean and standard deviations were calculated from the five remaining results.

For cytotoxicity assay, NIH3T3 cells were kept in a high glucose Dulbecco’s Modified Eagle Medium ([DMEM], Corning Inc., New York, NY, USA) supplemented with 10% (*v*/*v*) fetal bovine serum (Gibco, Gaithersburg, MD, USA) and 1% (*w*/*v*) antibiotic/antimycotic cocktail (Gibco, Gaithersburg, MD, USA) at 37 °C under 5% CO_2_. The cultured medium was replaced every 2–3 days and detached from a tissue culture plate by 0.2% trypsin–EDTA (Gibco) treatment for 2 min, followed by collection via centrifugation. The collected cells were seeded in 48-well plates at 10,000 cell/well and cultured for 24 h. The cocoons were irradiated by gamma-ray at (30 kGy) for sterilization and immersed in a serum-free medium at 1 g/10 mL, followed by 24 h incubation at 37 °C. For the extract treatment, the culture medium was replaced with the sample extract, and the cells were incubated for another 24 h. The cell survival rate was determined by a trypan blue exclusion assay. Briefly, the cells in each well were collected by trypsin–EDTA treatment and resuspended in Dulbecco’s phosphate-buffered saline ([DPBS], Corning Inc.), then mixed with an equal volume of trypan blue solution. The live/dead cells were counted, and the viability was measured using a cell counter (LUNA-FL; Logos Biosystems, Anyang, South Korea). The cell viability test was triplicated, and the viabilities were presented as mean ± standard deviation (SD). For statistical analysis, a one-way ANOVA was performed with a Tukey post-hoc test for multiple-group comparison after the Shapiro–Wilk normality test was conducted.

For statistical analysis of the degumming ratio (Figure 1) and the composition of the secondary structure of the silk (Figure 3), a one-way ANOVA or a Kruskal–Wallis test was performed with a Tukey post-hoc test or Dunn’s test for multiple-group comparison, respectively, according to the Shapiro–Wilk normality test result. The statistical significance levels were indicated at *p < 0.05.

## 4. Conclusions

In this study, cocoons and SFs were prepared from various *Bombyx mori* silkworm strains, and their structural characteristics and properties were examined. Depending on the silkworm strain, the degumming ratio, molecular conformation, solution viscosity, and mechanical properties of the silk were different. Considering the solution viscosity and mechanical properties, silkworm strain 9671 can be considered the best option to obtain silk with good physical properties. Regardless of the silkworm strain, all silkworm cocoons showed good cytocompatibility.

The results of this study indicate that various properties of silk with good cytocompatibility can be obtained by choosing the appropriate silkworm strain. Moreover, the appropriate silkworm strain might be a promising option for developing silk products for various applications, including in the biomedical and cosmetic fields. Therefore, it is necessary to focus on utilizing different silkworm strains and to conduct further studies on the topic.

## Figures and Tables

**Table 1:** The external features, SEM images, filament diameter, thickness, and weight of the silk cocoons produced from different silkworm strains. The magnification bar in the SEM images represents 200 μm.

Silkworm Strain	External Features of Cocoons	SEM Image		Filament Diameter (µm, Mean ± SD, *n* = 30)		Thickness of Cocoons (mm, Mean ± SD, *n* = 20)	Weight of the Silk Cocoons (Excluding the Pupae, G, Mean ± SD, *n* = 3)
		Outside of Cocoon	Degummed Silk	Outside of Cocoon	Degummed Silk		
9671	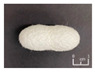	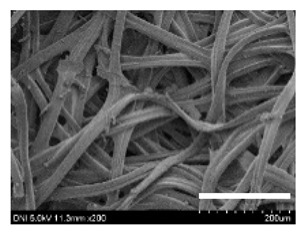	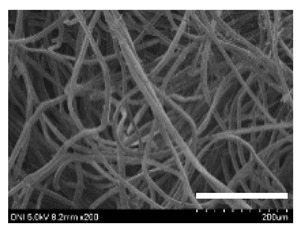	29.1 ± 2.7	15.3 ± 1.6	1.15 ± 0.17	0.49 ± 0.02
2607	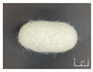	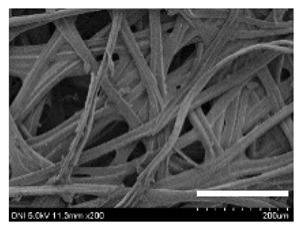	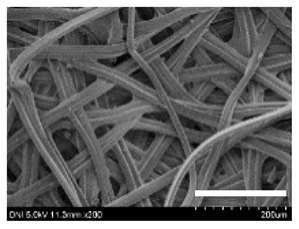	28.9 ± 3.0	14.5 ± 1.1	1.01 ± 0.14	0.45 ± 0.04
2203	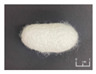	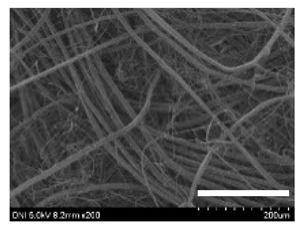	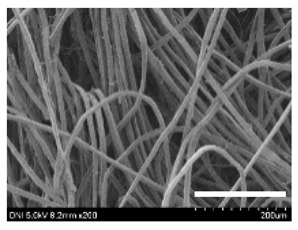	27.7 ± 2.7	14.1 ± 1.3	0.98 ± 0.11	0.41± 0.02
9153	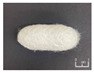	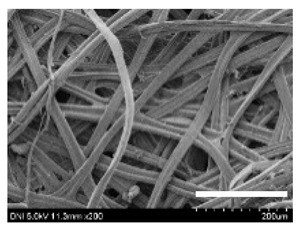	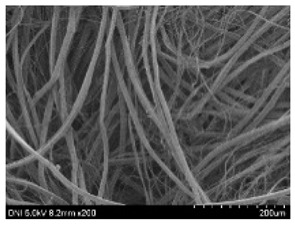	28.8 ± 2.6	14.5 ± 1.5	1.16 ± 0.10	0.44 ± 0.04
8013	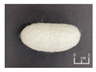	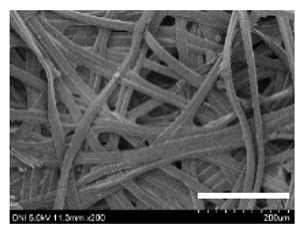	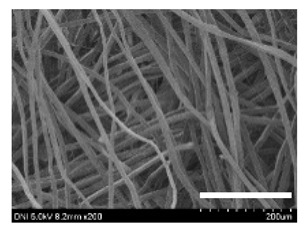	27.2 ± 2.6	13.7 ± 1.3	1.09 ± 0.20	0.39 ± 0.02
2301	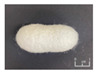	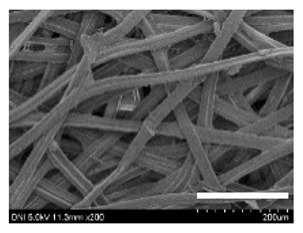	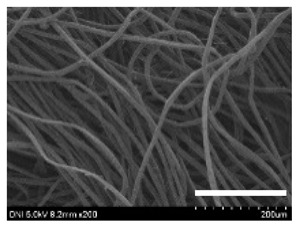	28.9 ± 2.4	14.7 ± 1.8	1.10 ± 0.17	0.40 ± 0.01
2603	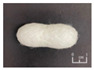	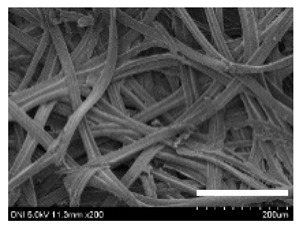	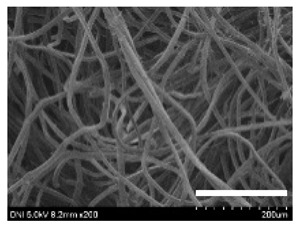	28.1 ± 2.7	13.1 ± 1.4	1.19 ± 0.12	0.43 ± 0.05
KJ5	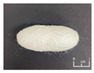	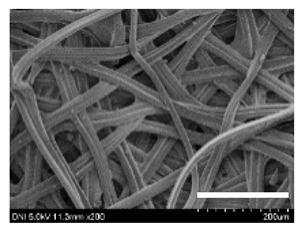	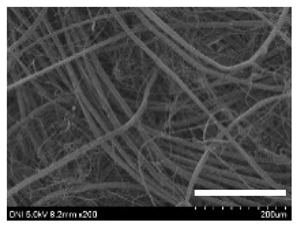	29.4 ± 2.5	13.2 ± 1.9	1.21 ± 0.16	0.44 ± 0.01
I-NOVI	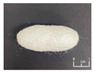	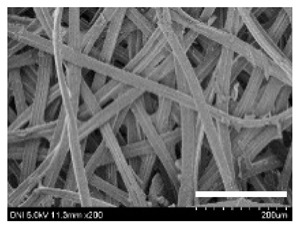	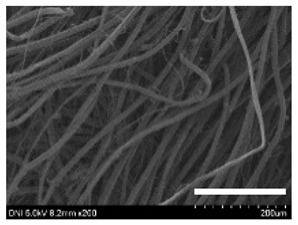	27.1 ± 2.1	13.9 ± 1.6	0.52 ± 0.09	0.20 ± 0.01
181	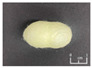	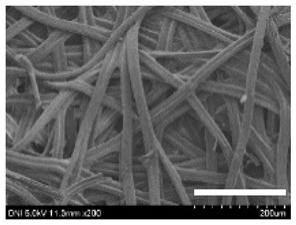	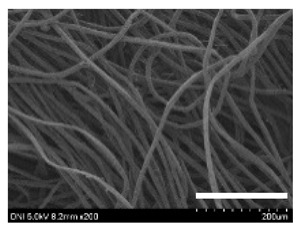	26.5 ± 2.4	12.4 ± 1.4	0.73 ± 0.19	0.13 ± 0.03

## Data Availability

The data presented in this study are available on request from the corresponding author.
